# Pulmonary valve replacement in patients with corrected tetralogy of Fallot

**DOI:** 10.15171/jcvtr.2017.12

**Published:** 2017-05-04

**Authors:** Fotios M. Mitropoulos, Meletios A. Kanakis, Christos Ntellos, Constantinos Loukas, Periklis Davlouros, Theophili Kousi, Andrew C. Chatzis

**Affiliations:** ^1^Department of Pediatric and Congenital Cardiac Surgery, Onassis Cardiac Surgery Center, Athens, Greece; ^2^Department of Cardiology, Tzaneio General Hospital, Piraeus, Greece; ^3^Department of Medical Physics, Medical School, National and Kapodistrian University of Athens, Greece; ^4^Department of Cardiology, University General Hospital of Patras, Greece

**Keywords:** Adult Congenital Heart Disease, Congenital Heart Surgery, Pulmonary Valve, Reoperation

## Abstract

***Introduction:*** Development of pulmonary insufficiency in patients with surgically corrected tetralogy of Fallot (TOF) may lead to severe right heart failure with serious consequences. We herein present our experience with pulmonary valve replacement (PVR) in these patients.

***Methods:*** From 2005-2013, 99 consecutive patients (71 males/28 females, mean age 38±8 years), underwent PVR after 7 to 40 (mean 29 ± 8) years from the initial correction. Seventy nine of the symptomatic patients presented in NYHA II, 14 in III and 2 in IV. All underwent PVR with a stented bioprosthetic valve, employing a beating heart technique with normothermic extracorporeal circulation support. Concomitant procedures included resection of aneurysmal outflow tract patches (n = 37), tricuspid valve annuloplasty (n = 36), augmentation of stenotic pulmonary arteries (n = 9), maze procedure (n = 2) and pulmonary artery stenting (n = 4).

***Results:*** There were 2 perioperative deaths (2%). One patient developed sternal dehiscence requiring rewiring. Median ICU and hospital stay was 1 and 7 days respectively. Postoperative echocardiography at 6 and 12 months showed excellent bioprosthetic valve performance, significant decrease in size of the right cardiac chambers and reduction of tricuspid regurgitation (TR) in the majority of the patients. At mean follow-up of 3.6 ± 2 years, all surviving patients remain in excellent clinical condition.

***Conclusion:*** Probability of reoperation for pulmonary insufficiency in patients with surgically corrected TOF increases with time and timely PVR by preventing the development of right heart failure is crucial for long-term survival. Current bioprosthetic valve technology in combination with the beating heart technique provides excellent immediate and short-term results. Further follow-up is necessary to evaluate long-term outcome.

## Introduction

Tetralogy of Fallot (TOF) is one of the commonest cyanotic congenital heart defects (CHD) and its treatment is considered as one of the success stories of modern medicine and surgery.^1^ Nevertheless, this is hampered by long-term morbidity due to right ventricular (RV) dysfunction secondary to pulmonary regurgitation (PR).^2^ Therefore, pulmonary valve replacement (PVR) is employed to prevent the detrimental effects of PR.^3^ Timely management although essential for optimal long-term functional and hemodynamic results, remains undetermined.^4^ We herein present our experience with PVR in patients with surgically corrected TOF using stented bioprosthetic valves.

## Patients and Methods

From September 2005 to December 2013, 99 consecutive patients, mean age 38 ± 8 (range 17-51) years, 71 males and 28 females with surgically corrected TOF underwent PVR after a mean time of 29 ± 8 (7-40) years from the initial surgical repair. All patients had undergone surgical correction of TOF with the transannular patch technique. Seventy-nine symptomatic patients presented in NYHA II, 14 in NYHA III and 2 in NYHA IV. Eighty-five patients underwent re-operation for the first time, while 9, 3 and 2 patients were re-operated for the second, third and fourth time respectively (Table 1).

**Table 1:** Enrolled patients’ data

**Parameters**	**Value**
No of patients	99
Gender	
Male	71
Female	28
Mortality	2/99 (2%)
Mean age (y)	38±8
Pre-operative NYHA status	
I	4
II	79
III	14
IV	2
No of reoperations	
1^st^	85
2^nd^	9
3^rd^	3
4^th^	2
Concomitant procedures	
Resection of aneurysmal RVOT patches	37
Tricuspid valve annuloplasty	36
Augmentation of stenotic PAs	9
Modified Maze procedure	2
Intraoperative PA stenting	4
CPB time (min)	47.5±12
Median stay (d)	
ICU	1
Hospital	7
Mean follow-up (y)	3.6±2

Abbreviations: NYHA, New York Heart Association; RVOT, right ventricular outflow tract; PA, pulmonary artery; ICU, intensive care unit; CPB, cardio-pulmonary bypass,

Patient referral and therefore study inclusion criteria were those set by Davlouros and colleagues: (*a*) asymptomatic patients with severe PR, progressive RV dilatation, and dysfunction and/or deterioration in exercise tolerance. (*b*) Symptomatic patients with established severe PR and RV dilatation regardless of RV function. (*c*) Patients with moderate to severe PR and associated lesions with significant hemodynamic impact requiring surgical intervention. (*d*) Patients with severe ventricular arrhythmias, associated with severe PR and RV dilatation irrespective of ventricular function.^5^ The only exclusion criteria applied were those conditions, which would diverge the procedure from the beating heart technique (e.g. performing a left side valve procedure or a residual VSD requiring cardioplegic arrest).

In all cases, the procedure was performed using the beating heart technique with extracorporeal circulatory support. Thorough preoperative echocardiographic evaluations determined RV dimensions and function and excluded the presence of intracardiac communications. Findings were confirmed by cardiac magnetic resonance imaging (MRI). Stented, oversized, third generation bioprosthetic valves (Aortic Magna -Edwards Life sciences, Soprano Armonia-Sorin and Mosaic - Medtronic Inc.) were implanted based on surgeon’s preference and availability. In addition, resection of aneurysmal outflow tract patches (n = 37), tricuspid valve annuloplasty (n = 36), augmentation of stenotic pulmonary arteries (n = 9), modified maze procedure (n = 2) and intraoperative pulmonary artery stenting (n = 4) were also performed (Table 1).

### Operative approach

**Figure 1:**
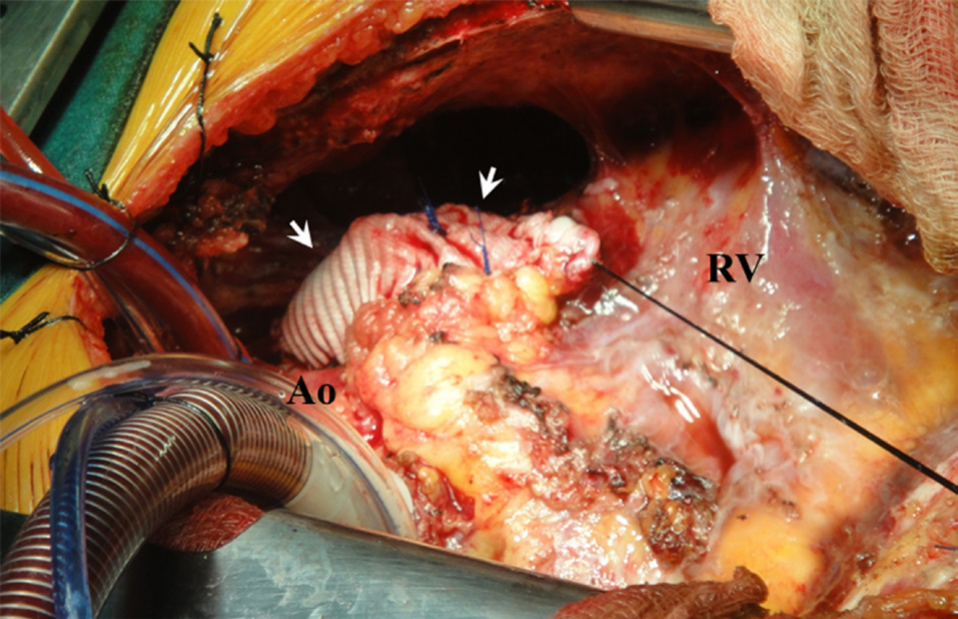
Arrows signify RVOT Dacron patch (surgeon’s view).

After induction of general anesthesia, redo midline sternotomy incision was performed with an oscillating saw. Cautiously all adhesions were meticulously removed by sharp dissection and electrocautery to achieve a dry field prior to heparinization. Standard bicaval or in some cases single right atrial to aorta cannulation was established and on occasion, arteriovenous (AV) femoral cannulation as necessary. Normothermic cardiopulmonary bypass was then established and the operation was accomplished using beating heart technique.^6^ The main pulmonary artery (MPA) was incised longitudinally and the old patch excised. A soft metal tip sucker was placed into the confluence of the branch pulmonary arteries and occasionally another one in the RV through the RV outflow tract (RVOT) to create a relatively dry operative field. Rudimentary pulmonary leaflets were excised. RV aneurysms or remaining subpulmonary muscle bands were resected as well. The MPA was thereafter reconstructed with a large piece of Dacron, where the valve would also be sewn on anteriorly, with the patch covering like a hood the MPA and the newly created RVOT. If necessary, the branch pulmonary arteries were enlarged with the use of autologous or bovine pericardium and occasionally with the aid of an intraoperatively inserted pulmonary artery stent. The largest suitable and available bioprosthetic valve was then sutured to the pulmonary annulus with a continuous polypropylene suture technique (Figure 1). Whenever appropriate, i.e. in any patient with 2+ or greater tricuspid regurgitation (TR), the TV was also repaired using various annuloplasty techniques.

### Patient follow-up

All patients were placed postoperatively on antiplatelet therapy with aspirin for 6 months. Periodic follow-up included clinical assessment, electrocardiography and transthoracic echocardiography at 6-month intervals. None of the patients required postoperative cardiac catheterization. Clinical assessment involved exercise capacity and detection of symptoms and signs of right heart failure. ECG study included QRS complex duration values. MRI was reserved for those patients with evidence of RV function deterioration.

At echocardiography, RV dimensions were assessed both qualitatively and quantitatively. Qualitative assessment of RV size was accomplished by calculation of RV area and mid-cavity diameter at end diastole, from the apical four chamber view wherein they should normally be smaller than those of the LV. In case of moderate RV enlargement, the ventricular cavity area matches that of the LV and shares the apex of the heart. Progression, however, of RV dilatation results in further increase of the cavity area surpassing therefore that of the LV and dominating the formation of the apex.^7^ RV function was assessed by means of tricuspid annular plane systolic excursion (TAPSE), measuring the level of systolic excursion of the lateral tricuspid valve annulus towards the apex in a four chamber view. In addition, tissue Doppler imaging (TDI) was used as a quantitative assessment of RV systolic and diastolic function by calculating myocardial velocities.^8^ TR was evaluated in a semi-quantitative manner by means of proximal isovelocity surface area (PISA) radius and vena contracta width. A vena contracta width ≥7 mm suggests severe TR, whereas a diameter <6 mm refers to mild or moderate TR.^9,10^ PR was assessed by jet size, deceleration rate and regurgitant fraction.^9,10^

### Statistical analysis

Preoperative and postoperative continuous variables were compared by paired *t* test. The significance of differences between two groups was assessed by Student’s *t* test. All results were expressed as mean ± standard deviation and a *P* value of <0.05 was considered statistically significant.

## Results

Total cardiopulmonary bypass time was 47.5 ± 12 minutes. There were two early deaths (2%). Both patients were in NYHA IV status preoperatively with severe RV dilatation and dysfunction. This was their fourth re-operation and they eventually died from multiple organ dysfunction syndrome (MODS). In particular, the first patient developed septic shock and died 45 days after surgery while the second severe coagulopathy due to hepatic failure and died 30 days postoperatively.

Of the surviving patients (n = 97), 5 had a cardioverter defibrillator implanted for sustained severe ventricular arrhythmias. Both patients who underwent the modified Maze procedure remained in sinus rhythm. One patient required rewiring for sternal dehiscence. Median ICU and hospital stay was 1 and 7 days respectively.

Follow-up period ranged from 6months to 8 (3.6 ± 2) years during which none of these patients required re-operation. All of them experienced significant clinical improvement and remain in excellent clinical condition.

### ECG assessment

**Figure 2:**
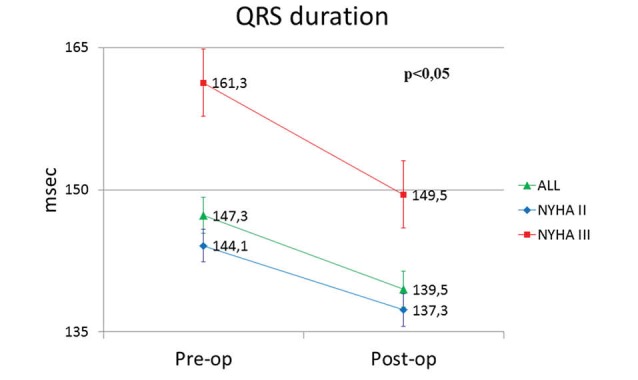
Significant postoperative reduction in QRS duration (*P* < 0.05)

QRS complex duration was significantly reduced from 147.3 ± 13.6 ms preoperatively to 139.5±13 ms postoperatively (p<0.05). Especially, in NYHA II patients, QRS duration significantly decreased postoperatively from 144.1 ± 11.4 ms to 137.3 ± 10.5 ms (*P *< 0.05) and in NYHA III from 161.3 ± 8.9 ms to 149.5 ± 9.7 ms (*P *< 0.05) respectively (Figure 2).

### Functional class

**Figure 3:**
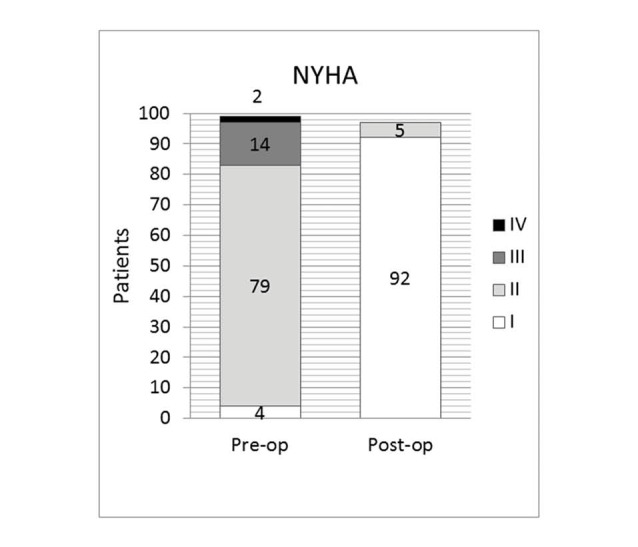
Dramatic clinical improvement in the vast majority of patients.

Significant improvement in NYHA status was achieved in the vast majority of the surviving patients. In particular, 92 patients are in NYHA I and 5 in NYHA II (Figure 3).

### Right ventricle

#### RV dimensions

**Figure 4:**
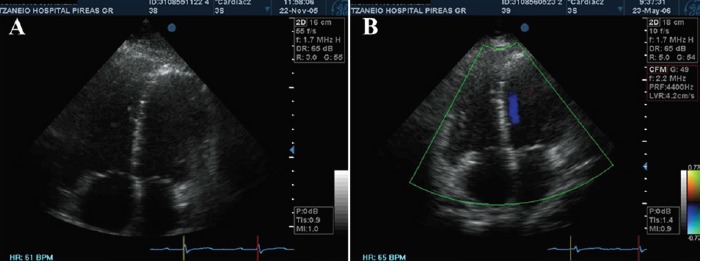
Comparison of preoperative (A) and postoperative (B) echocardiogram in a patient with RV dilatation depicting the postoperative decrease in RV dimensions.

At qualitative assessment, RV dilatation was found to improve from severe in 17 patients to moderate in 10 and mild in 7 patients respectively (Figure 4). The other 82 patients improved from moderate to mild dilatation.

**Figure 5:**
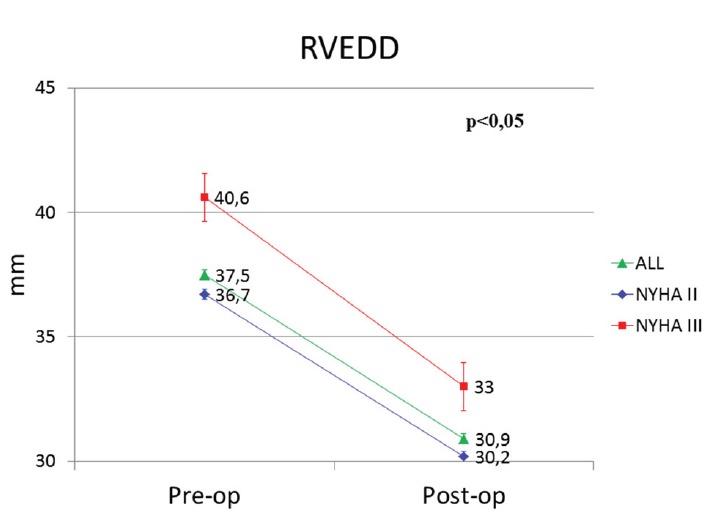
Significant postoperative reduction in RV end diastolic diameter (*P* < 0.05).

Quantitative study showed a significant decrease in RV end diastolic diameter (RVEDD) from 37.5 ± 2.8 mm to 30.9 ± 2.8 mm postoperatively (*P *< 0.05). More specifically, in patients who preoperatively were in NYHA II and III status, RVEDD decreased significantly (*P *< 0.05) from 36.7 ± 1.3 mm to 30.2 ± 1.3 mm and from 40.6 ± 1.7 mm to 33.0 ± 3.1 mm respectively (Figure 5). It should be noted that RV dilation remained severe in the two patients who finally died.

#### RV dysfunction

**Figure 6:**
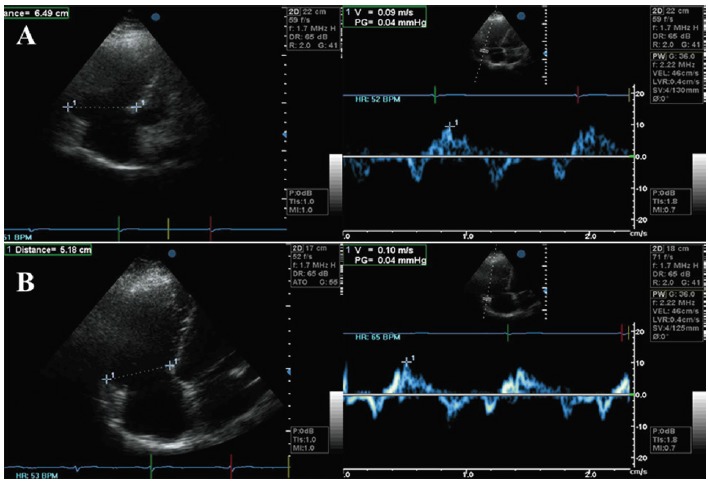
Echocardiographic study in a patient with preoperative RV dysfunction (A): RV dysfunction remains unchanged after PV replacement with reduced, nevertheless, RV dilatation (B).

RV dysfunction was present in 12 patients preoperatively, 2 of which eventually died. Of the 10 surviving patients, 7 persisted with RV dysfunction (preoperative: TAPSE 8.14 ± 2.67 mm, TDI<11.5 cm/s, postoperative: TAPSE 10.85 ± 4.22 mm, TDI <11.5 cm/s) yet with improved (moderate) RV dilatation. Of these, 6 patients in preoperative NYHA III status are now in NYHA II, whereas 1 patient remained in NYHA II status (Figure 6).

**Figure 7:**
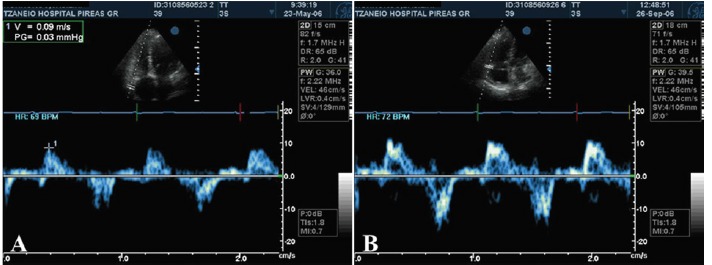
Echocardiographic study in a patient with preoperative RV dysfunction (A) who recovered RV function 6 months after the operation (B).

Three patients, however, recovered RV function (preoperative: TAPSE 14.66 ± 0.58 mm, TDI <11.5 cm/s, postoperative: TAPSE 17.0 ± 1.0 mm, TDI >11.5 cm/s) 6 months after the operation. All of them were in NYHA III with severe RV dilation preoperatively and improved to NYHA I status in spite of, moderate, although improved, RV dilatation (Figure 7).

#### Tricuspid regurgitation

**Figure 8:**
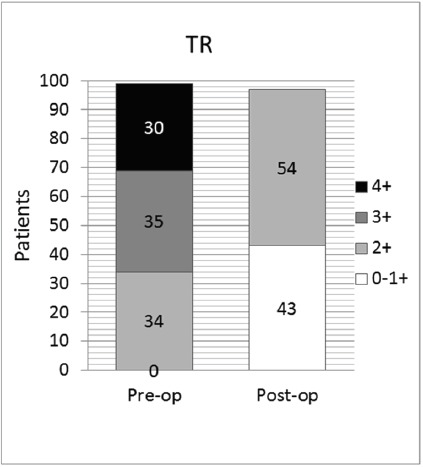
Postoperative reduction in tricuspid regurgitation.

Preoperative TR was evaluated as severe, moderate and mild in 30, 35 and 34 patients respectively. The tricuspid valve was repaired using various annuloplasty techniques (Kay, pericardial strip, Kalangos ring, conventional rings) in 36 patients, all 30 with severe and selected 6 with moderate regurgitation. All patients had mild or less TR at follow-up (Figure 8).

#### Pulmonary regurgitation

All patients (n = 99) had severe PR prior to operation. Following PVR, all surviving patients showed excellent prosthetic pulmonary valve function with only 11 of them having mild regurgitation, while the remaining 88 an absolutely competent prosthesis. Postoperative mean pulmonary valve gradient was 9 ± 2 mm Hg.

## Discussion

Longstanding PR has been recognized to have deleterious effects on RV function. Indeed, progressive RV volume overload results in severe late complications. Exercise limitation, right and left ventricular dysfunction, electrocardiographic abnormalities and most importantly, development of life threatening atrial and ventricular arrhythmias are the most common.^11^ It seems that the main cause of sudden death in these patients is fatal arrhythmias resulting from RV dysfunction and therefore preservation or restoration of RV function may reduce the risk.^2,11-13^ Nevertheless, in the case of established supraventricular arrhythmias, a combined procedure with cryoablation seems beneficial.^5^

Some degree of PR is almost always present in patients following anatomical correction of TOF. Pulmonary regurgitation is well tolerated for years, yet the chronic effects on RV function may be, dramatic.^14^ Patients often are unaware of any symptoms, until RV dysfunction becomes severe. In addition, for undetermined reasons, a right to left ventricular interaction ensues with subsequent left ventricular dysfunction.^14^ Pulmonary regurgitation is frequently underestimated on physical examination, since the anticipated diastolic murmur is often soft and short due to rapid equalization of the diastolic pressures in pulmonary artery and right ventricle. The regurgitant jet is also often missed on two-dimensional echocardiography due to low velocity and laminar flow pattern. Therefore, all patients with previous surgical repair of TOF should undergo routine monitoring to determine changes in cardiothoracic index followed by a comprehensive echocardiographic examination.^14^

Currently, PR secondary to valve commissurotomy, transannular enlargement and patching presents as the most common finding and subsequent indication for reoperation in patients with repaired TOF. PVR is consequently considered for preservation of the jeopardized RV function. Yet, although the importance of chronic RV volume overload is well recognized, the ideal time for PVR remains a debatable issue.^15^

Appropriate and timely management of postoperative PR remains essential for beneficial long-term functional and hemodynamic results. Following PVR, subjective clinical improvement has been reported in several studies. Objective improvement in RV function and reduction in RV size, subsequent to PVR, has also been shown.^15-22^ Bove and colleagues reported, in a group of 11 patients, favourable change in RV size by demonstrating significant reduction in cardiothoracic index as also diminished echocardiographic right to left ventricular end diastolic dimensions ratio.^18^ Ilbawi et al displayed a significant reduction of the cardiothoracic index in 42 and a decrease in angiographically determined RV end systolic volumes in 18 patients.^19^ Warner and colleagues reported a 30% reduction in echocardiographic RV end diastolic diameter (RVEDD) in 16 patients after PV replacement for PR.^11^ All the aforementioned studies also documented improvement in exercise tolerance.

Pulmonary valve replacement should be considered before the development of irreversible RV dysfunction and can be performed with low operative risk (1%-2%).^2,11,12^ Evidence suggests that delayed intervention leads to disastrous consequences.^14^ Although subjective improvement in clinical symptoms may occur after delayed re-operation, RV function and volumes often remain unchanged as chronic myocardial exposure to severe PR results in irreversible contractile impairment.^17^

Early detection of TR may signify and prove reliable indicator of the appropriate timing for PVR and subsequent RV function preservation.^2,15^ Davlouros and colleagues classified the indications for PV replacement based on clinical and PR and RV dilatation assessment criteria.^5^ These constitute our current surgical indications; patients undergoing surgery in extension of these criteria exhibited varied outcomes, with some of them, nonetheless, experiencing significant clinical improvement. Therrien et al concluded that PVR should be undertaken before RV end-diastolic volume reaches 170 mL/m^2^ or RV end-systolic volume reaches 85 mL/m^2^ to increase the chances of normal RV volume restoration after repair.^17^ In a recent study, Dave and colleagues showed that timely insertion of a PV substitute in young patients, when RV end-diastolic volume exceeds 150 mL/m^2^, is directly associated with improvement in RV dimension and function, in a 6 month period.^21^ The duration of the QRS complex is directly proportional to RV dimensions and right bundle branch block is anticipated in almost 95% of patients. Therefore, QRS duration may also designate the time of re-operation, although clear limits are yet to be defined.^2,14^

The ideal valve for the pulmonary position is yet to be found. Selected PV prostheses should demonstrate optimal hemodynamics, durability, easy implantation and, not the least, at a relatively low cost. A variety of valves have been used over the years for PVR and include mechanical, xenografts (stented or stentless), homografts, autologous pericardial valves and more recently bovine jugular valves (stented or stentless). The use of mechanical valves in the pulmonary position has been reported, but has significant drawbacks, largely due to the frequent occurrence of thromboembolic phenomena and valve failure.^15,22^

Earlier results with stented xenografts were disappointing due to premature deterioration and calcification and reported freedom from reoperation of only 37% at 5 years.^24^ Fortunately, homografts came around and became the ‘conduit of choice’ for the pulmonary position. However, they also deteriorate with time and actuarial freedom from reoperation at 5 and 10 year varies from 74%-85% and 54%-69% respectively.^25,26^ During the last decade bioprosthetic valve technology has made some distinct advances. Third generation valves share some unique characteristics that include glutaraldehyde zero pressure fixation and treatment with alpha amino oleic acid (AOA), an anti-mineralization agent that has been shown to reduce leaflet calcification in animal models.^27^ These techniques have significantly increased the durability of these valves. Over the years, in an effort to achieve optimal hemodynamics with long-term durability, our approach to patients requiring PVR has evolved into the following strategy: employment of third generation, stented, oversized bioprosthetic valves.

Although Kanter and colleagues have reported excellent short-term results with the use of a stentless aortic valve we prefer the stented counterpart since with oversizing the stented framework minimizes the compression from the sternum after closure.^28^ This is supported by the low incidence of postoperative insufficiency or stenosis in our group of patients who received oversized valves. The other theoretical advantage of using oversized valves is to minimize the RV to pulmonary artery gradient (albeit functioning in a low pressure system) and the high pressure effect that cause long-term structural dysfunction. With reduced diastolic trans-prosthetic pressure gradient and low closing stress in the pulmonic position the, in any case, limited mechanical destruction is even further minimized.^29^ Also, we have placed these patients on antiplatelet therapy with aspirin for 6 months until endothelialization has occurred, extrapolating from the existing data with the use of these valves in adults with acquired valve disease. Although, the use of percutaneously implanted bovine jugular valves is still in its infancy and long term studies are warranted to determine its efficacy, safety and durability, the stented valves we have used provide the necessary setting for a possible future intervention of this kind.^15,30^

## Conclusion

Although the beating heart approach is technically more demanding, it has the significant advantage of avoiding myocardial ischemia/reperfusion syndrome which occur during cardiac arrest. As a result we did not observe any patients with low cardiac output syndrome postoperatively. A word of caution though for the beating heart technique; preoperative work up should exclude any intracardiac communication to avoid the complication of air embolism, which can be devastating. The incidence of serious postoperative complications in our series was low and none of these patients had clinical evidence of infective endocarditis during the study period.

Patients with surgically corrected TOF require clinical and echocardiographic evaluation on a regular basis in order to detect and follow the progression of PR. Optimal timing of PVR remains a subject of debate. It is highly important to identify the time span that the RV can endure PR before irreversible damage develops (not too late) and avoiding an untimely re-operation (not too early).

In experienced centers, PVR is achieved with low morbidity and mortality (especially with the beating heart technique) and should be accompanied by a surgical strategy to optimize hemodynamic performance and extend durability of the valve. Our findings suggest that currently available bioprosthetic valves in the pulmonary position provide excellent immediate and intermediate results. Longer follow-up is necessary to determine the long-term performance of these valves.

## Limitations

As a retrospective study bears its well-known limitations.

## Ethical issues

Not applicable.

## Competing interests

The authors declare no conflict of interest regarding this study.
